# Vitamin D and Vitamin D‐binding protein and risk of bladder cancer: A nested case‐control study in the Norwegian Janus Serum Bank Cohort

**DOI:** 10.1002/cam4.3960

**Published:** 2021-06-03

**Authors:** Helga H. Hektoen, Trude E. Robsahm, Jo S. Stenehjem, Karol Axcrona, Ronnie Babigumira, Alison M. Mondul, Randi E. Gislefoss, Bettina K Andreassen

**Affiliations:** ^1^ Department of Research Cancer Registry of Norway Oslo Norway; ^2^ Department of Biostatistics Oslo Centre for Biostatistics and Epidemiology University of Oslo Oslo Norway; ^3^ Department of Urology Akershus University Hospital Lørenskog Norway; ^4^ Department of Epidemiology University of Michigan School of Public Health Ann Arbor MI USA

**Keywords:** bladder cancer risk, cancer risk, case–control study, vitamin D, vitamin‐binding protein

## Abstract

**Background:**

High circulating levels of vitamin D (25(OH)D) are suggested to reduce the risk of urinary bladder cancer (BC), but the evidence is weak, and several studies lack sufficient adjustment for potential confounders (e.g., smoking, body mass index (BMI), and physical activity). Moreover, few studies have investigated the role of vitamin D‐binding protein (DBP) in this context. We conducted a matched nested case–control study including 378 cases and 378 controls within the Norwegian population‐based Janus cohort, using serum collected 5–41 years prior to diagnosis, to study 25(OH)D and BC risk, by taking circulating DBP into account.

**Methods:**

Cox regression models were used to estimate hazard ratios (HRs) and 95% confidence intervals (CIs), for 25(OH)D, DBP, and the molar ratio of 25(OH)D:DBP, an estimate of unbound (free) 25(OH)D levels. We adjusted for smoking (status and pack‐years), BMI, physical activity, education and (mutually) for 25(OH)D and DBP. Restricted cubic splines were employed to examine nonlinear associations.

**Results:**

High optimal levels of circulating 25(OH)D (≥100 nmol/L) (HR 0.35, 95% CI 0.19–0.64) were associated with decreased BC risk, when compared with insufficient concentrations (50–74 nmol/L). This association was less pronounced for optimal levels (75–99 nmol/L) (HR = 0.69, 95% CI 0.47–1.01). Moreover, estimated free 25(OH)D, was associated with decreased BC risk for molar ratio 17–21 (HR 0.66, 95% CI 0.44–0.97) and ≥22 (HR 0.50, 95% CI 0.29–0.82), compared to molar ratio 11–16. The HR function for BC risk was not linear, rather reversed u‐shaped, with the highest HR at 62.5 nmol/L and 13.5 molar ratio, respectively.

**Conclusion:**

High levels of total and estimated free 25(OH)D were associated with reduced risk of BC, compared with insufficient concentrations. DBP was not associated with BC risk. We did not observe any impact of DBP or any of the studied lifestyle factors on the association between 25(OH)D and BC.

## Graphical abstract

In this large prospective case‐control study nested in a population‐based cohort, we found that high circulating levels of 25(OH)D, both total and free concentrations, were associated with reduced risk of bladder cancer, compared with insufficient levels. Vitamin D binding protein was not associated with bladder cancer risk.

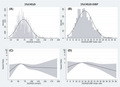

## 1 INTRODUCTION

Urinary bladder cancer (BC) is the most common genitourinary malignancy after prostate cancer worldwide.^1^ The incidence rates are 3–4 times higher in men than in women and BC risk is increasing with increasing age.^2^, ^3^ The main alterable risk factors for BC are smoking and exposure to chemicals.^4^, ^5^, ^6^ Other suggested risk factors include lifestyle‐related factors such as body mass index (BMI), blood pressure, physical activity, and various dietary and nutritional factors, including vitamin D.^7^, ^8^, ^9^

Vitamin D is synthesized in the skin by ultraviolet radiation from the sun, or obtained from food and supplements, and must undergo activation through two steps to become the biologically active hormone; first to form 25‐hydroxyvitamin D (25(OH)D) in the liver and then to form 1–25‐dihydroxyvitamin D (1,25OH_2_D) in the kidney.^10^ The active hormonal form of vitamin D is vital for maintaining bone health, but does also regulate several other biological functions, including mechanisms involved in carcinogenesis, such as cell growth and differentiation.^11^ Various preclinical studies have shown that 1,25(OH)_2_D can suppress tumor progression in BC and other cancers.^12^ For example, in a study performed on rats, Konety et al., found that 1,25(OH)_2_D inhibited BC tumorigenesis and cell proliferation.^13^

25(OH)D is the primary circulating form of vitamin D, and is considered the best indicator of an individual's vitamin D status.^14^ The majority of circulating 25(OH)D is bound to vitamin D‐binding protein (DBP) (~88%) and albumin (~12%) and only a small proportion remain unbound (0.03%).^15^, ^16^ Most laboratory assays do not differentiate between the bound and the unbound (free) state, but a proxy of the free state can be estimated by the molar ratio of the total 25(OH)D to DBP, which is considered a reasonable measure of biologically available 25(OH)D.^17^ In associations with cancer risk, it is unknown whether the total or the free state is more relevant to study.

Several observational studies report associations between circulating 25(OH)D concentrations and cancer risk at various sites, including BC.^18^ The most recent meta‐analysis on circulating 25(OH)D levels and BC risk, found a reduced risk of BC with higher concentrations of 25(OH)D.^19^, ^20^ However, the individual studies did not report a clear association, and they vary according to adjustment for factors such as smoking history, BMI, and physical activity, which are related to the levels of 25(OH)D.^21^, ^22^ Moreover, few studies have investigated the potential role of DBP, which is suggested to modify the association between 25(OH)D and BC risk.^23^

In this study, we used stored serum from the population‐based Janus Serum Bank Cohort (Janus Cohort) to examine total and free 25(OH)D as well as circulating DBP in relation to subsequent BC risk. We also examined potential interactions with smoking, BMI and physical activity.

## 2 METHODS

### 2.1 Study population

The Janus Serum Bank Cohort is a population‐based biobank containing serum samples from 292,851 Norwegian men and women who participated in one of five large health surveys conducted between 1972 and 2004. Participants were aged 35–49 years at recruitment. Detailed descriptions of the cohort and the data available have been published elsewhere.^24^, ^25^ Our study was nested within the Janus Cohort and approvals for the study were obtained from the Janus Serum Bank Board and from the Regional Committee for Medical Research Ethics.

### 2.2 Identification of cancer cases and controls

The Cancer Registry of Norway (CRN) has been required by law to record cancer diagnoses since 1953, and holds complete and high quality data.^26^ BC cases in the Janus Cohort were identified by linkage to the CRN and were required to be 1) histologically verified BCs of the transitional cell type (morphological codes: 8120, 8130 and 8131, according to International Classification of Disease for Oncology, 3rd revision), 2) without any previous cancer diagnosis (except basal cell carcinoma), and 3) diagnosed a minimum of 5 years after blood draw (recruitment). The selection of BC cases consisted of high‐graded Ta, carcinoma in situ (Tis), tumors invading lamina propria (T1), and tumors invading muscularis propria and further (T‐stage T2–T4), thus excluding low‐graded noninvasive tumors (Ta).

Follow‐up began at recruitment into the Janus Cohort between 1972 and 2003 and continued until the date of BC diagnosis, emigration, death or the end of follow up at 31 December 2016, whichever came first. During follow‐up, a total of 1058 BC cases were identified (using the abovementioned case criteria). The number of included cases were limited to a random selection of 400 BC patients, based on statistical power and laboratory cost considerations.^25^

Controls were required to be resident in Norway, alive and without a cancer diagnosis before index date (date of BC diagnosis of the associated case). One control was sampled at random with replacement (incidence‐density sampling) and matched to each case (1:1 case–control ratio). The control was matched to each case on sex, year of birth, date of blood draw, season of blood draw within the following 3‐month intervals within the same calendar year (December‐February, March‐May, June‐August, September‐November), and county of blood draw. A flow chart of the study design and exclusions is presented in Figure S1.

### 2.3 Vitamin D and Vitamin D‐binding protein

Serum was collected from nonfasting subjects, and stored at −25⁰C. Serum concentrations of 25(OH)D and DBP were measured at the National Hormone Laboratory, Oslo University Hospital, participants of the vitamin D External Quality Assessment Scheme that ensures analytical reliability of 25(OH)D. Serum concentrations of 25(OH)D were measured by a liquid chromatography/tandem mass spectrometry method and DBP by a radioimmunoassay (both assays are developed at the Hormone Laboratory). The matched case–control sets were analyzed within the same batch. A blinded quality control sample was included in each of the 25 batches. The interassay coefficient of variation (CV) was 11.1% at 60.1 nmol/L for 25(OH)D, and 10.5% at 6.5 µmol/L for DBP, respectively.

Since 25(OH)D concentrations are strongly affected by season, we used season‐adjusted concentrations in our analysis, in addition to matching case and controls on date of blood draw.^27^ We modeled the seasonal variation in our study sample by performing a least square fit of a sine function to the measured concentrations of 25(OH)D versus date of blood draw. An additional file shows this method more in detail (see supplementary including Figure S2).

The categories of 25(OH)D were defined based on previously defined clinical cut points by the endocrine society ^14^; deficient <50 nmol/L, insufficient (50–74 nmol/L), optimal (75–99 nmol/L), and high optimal (≥100 nmol/L), with 50–74 nmol/L being the reference category reflecting the average level in the Norwegian population.^28^

The molar ratio of 25(OH)D:DBP was used as an estimate for free circulating 25(OH)D, which previously has been described as a valid approximation of “free 25(OH)D”.^17^ The molar ratio is a simplification of the equilibrium equation between free and bound 25(OH)D, which neglect the contribution from albumin.^15^, ^17^

To be consistent throughout the paper, we used the clinical categories of 25(OH)D as guidance when categorizing DBP and 25(OH)D:DBP. More specifically, we applied the percentiles of the four clinically defined cut‐points of 25(OH)D to the distribution of DBP and 25(OH)D:DBP leading to the following categories for DBP µmol/L (<3.9, 3.9–4.5, 4.6–5.4, and ≥5.4) and 25(OH)D:DBP molar ratio x 10^3^(<11, 11–16, 17–21, and ≥22).

### 2.4 Covariates

All individuals in the Janus Cohort underwent health examinations and filled out health related questionnaires.^25^ Information about smoking history was based on questionnaire data, and contained smoking status (never, former, and current smokers), and duration and intensity of smoking. Pack‐years were calculated by multiplying number of packs smoked per day with number of years smoked. We created a smoking variable consisting of five categories (never smokers, former smokers, and current smokers in three categories of pack‐years (tertiles)). Height and weight were measured by trained health personal. BMI was calculated and categorized according to the World Health Organization's classification: underweight and normal weight (<25 kg/m^2^), overweight (25–29.9 kg/m^2^), and obese (≥30 kg/m^2^). Information about physical activity was obtained from the questionnaires, categorized as sedentary, moderately active, and active. The health examination also included measurement of blood pressure, cholesterol, and triglycerides.

Information about occupation and education was obtained from Statistics Norway. Occupational working titles were categorized as high risk (yes or no) and was based on existing knowledge about chemical exposures in certain occupations that previously has shown to be related to BC risk.^7^, ^29^, ^30^ More details about the categorization of high risk occupations are published previously.^7^ Education was categorized as unknown, compulsory, upper secondary and college/university.

### 2.5 Statistical analysis

Descriptive statistics were used for patient characteristics. Stratified Cox regression was used to estimate hazard ratios (HRs) with 95% confidence intervals (CIs) of BC risk for four categories of 25(OH)D and DBP, and the molar ratio of 25(OH)D:DBP. Moreover, to explore the underlying shape of the effect of interest, the HR was modeled as restricted cubic splines with 4 knots dependent on 25(OH)D and 25(OH)D:DBP, using the STATA package rscgen. The knots were, following Harrell, placed at the 5th, 35th, 65th, and 95th percentiles.^31^ A likelihood ratio test was applied to compare the fit of the linear vs. the spline models.

In addition to be conditioned on the matching factors (age, sex and date, season, and county of blood draw) in model 1, the multivariable analyses were adjusted for smoking, BMI, physical activity and education (model 2). Model 3 included in addition mutual adjustment for DBP and 25(OH)D. Occupation, blood pressure, cholesterol, and triglycerides were all entered into the multivariable model to evaluate their impact on the risk estimates of DBP, 25(OH)D, and 25(OH)D:DBP. However, they were not associated with BC risk (LR test, *p* > 0.2) and did not change the risk estimates of interest on BC risk (HR) more than 10%, and were thus not included in the final models.

As smoking, BMI, physical activity, and DBP are hypothesized to be effect modifiers of the association between 25(OH)D and BC risk, we conducted analyses stratified by these variables, and tested for interaction. Statistical interaction was evaluated using the likelihood ratio test.

To assess the influence of extreme values of 25(OH)D and DBP concentrations on the results, we performed sensitivity analyses excluding persons with values below the 2.5 percentile or above the 97.5 percentile, which did not influence the results (see Table S1 and Figure S3). All statistical analyses were performed using STATA version 15.1 (StataCorp, College Station, TX). The statistical significance level was set to 5%.

## 3 RESULTS

Distribution of population characteristics are presented for both cases and controls in Table 1 and stratified for previously defined 25(OH)D categories in Table 2. Characteristics of cases and controls were comparable for most of the variables except for smoking with a larger proportion being current smokers among cases (59%), compared to controls (42%), in addition to higher mean of pack‐years among cases. Median concentrations of 25(OH)D were slightly lower among cases 68.3 (54.8–82.9) than among controls 71.7 (53.8–88.0). Table 2 shows that individuals with 25(OH)D deficiency (<50 nmol/L) tended to be heavier smokers, have a higher BMI and were less physically active, compared with individuals with higher 25(OH)D concentrations. Median concentrations of DBP and consequently molar ratio of 25(OH)D:DBP increased with increasing 25(OH)D concentrations.

**TABLE 1:** Baseline characteristics by case–control status

Characteristics	Case	Control
Male, n (%)	320 (85)	320 (85)
Female, n (%)	58 (15)	58 (15)
Year of birth, median (range)	1936 (1928–1946)	1936 (1928–1946)
Season of blood draw, n (%)		
Darker season (November–April)	179 (47)	169 (44)
Sunnier season (May–October)	199 (53)	209 (55)
Smoking status, n (%)		
Never smoker	74 (20)	100 (26)
Former smoker	80 (21)	119 (32)
Current smokers	224 (59)	159 (42)
Pack‐years, mean (SD)	18.0 (9.7)	13.2 (7.8)
BMI (kg/m^2^), mean (SD)	24.8 (3.1)	24.9 (3.0)
BMI (kg/m^2^), n(%)		
Normal (<25)	208 (55)	215 (57)
Overweight (25–29)	148 (39)	139 (37)
Obese (≥30)	22 (6)	24 (6)
Physical activity		
Sedentary	78 (21)	65 (17)
Moderately active	217 (57)	222 (58)
Active	83 (22)	91 (24)
Hypertension		
No	213 (56)	208 (55)
Yes	165 (44)	170 (45)
High risk occupation, n (%)		
No	251(66)	269 (71)
Yes	115 (30)	100 (26)
Unknown	12 (3)	9 (2)
Education, n (%)		
Compulsory	137 (36)	144 (38)
Upper secondary	186 (49)	180 (48)
College/University	55 (15)	54 (14)
Time between blood draw and diagnosis (years), median (range)	22 (16–28)	
25‐hydroxyvitamin D(nmol/L), median (range)	68.3 (54.8–82.9)	71.7 (53.8–88.0)
Vitamin D‐binding protein (DBP) (µmol/L), median (range)	4.5 (4.1–5.0)	4.5 (4.0–4.9)
25(OH)D:DBP molar ratio (x 10^3^)	15.2 (12.0–18.4)	15.7 (12.1–19.8)
Cholesterol (mmol/L), median (range)	6.0 (5.2–6.8)	6.1 (5.3–6.9)
Triglycerides (mmol/L), median (range)	1.5 (1.2–2.3)	1.7 (1.2–2.2)

Abbreviations: 25(OH)D, 25‐hydroxyvitamin D; BMI, body mass index; DBP, Vitamin D‐binding protein; SD, standard deviation.

**TABLE 2:** Baseline characteristics by clinical cut points of 25(OH)D

	25(OH)D (nmol/L)			
	Deficient	Insufficient	Optimal	High Optimal
Characteristics	<50	50–74	75–100	≥100
Age	44 (6.2)	45 (8.3)	44 (7.2)	43 (7.5)
Sex				
Male, n(%)	107 (83)	272 (84)	185 (85)	76 (88)
Female, n(%)	22 (17)	52 (16)	32 (15)	10 (12)
Smoking status, n (%)				
Never smoker	23 (17)	84 (25)	47 (30)	22 (25)
Former smoker	32 (24)	86 (26)	67 (30)	20 (23)
Current smokers	74 (57)	157 (48)	108 (50)	44 (51)
Pack‐years, mean (SD)	17.2 (10.1)	16.5 (9.2)	13.8 (8.3)	15.6 (8.4)
BMI (kg/m^2^), mean (SD)	25.4 (3.5)	24.9 (3.0)	24.7 (2.9)	24.3 (2.3)
BMI (kg/m^2^), n(%)				
Normal (≤25)	63 (47)	179 (53)	135 (60)	56 (63)
Overweight (25–29)	57 (43)	133 (40)	77 (34)	29 (32)
Obese (≥30)	12 (9)	20 (6)	13 (5)	3 (3)
Physical activity				
Sedentary	42 (33)	58 (18)	32 (15)	11 (13)
Moderately active	67 (52)	196 (60)	129 (59)	47 (55)
Active	20 (16)	70 (22)	56 (26)	28 (33)
Hypertension				
No	67 (52)	183 (56)	119 (55)	52 (60)
Yes	62 (48)	141 (44)	98 (45)	34 (40)
High risk occupation, n (%)				
No	84 (65)	218 (67)	153 (71)	65 (76)
Yes	39 (30)	99 (31)	61 (28)	16 (19)
Education, n (%)				
Compulsory	57 (44)	109 (34)	81 (37)	34 (40)
Upper secondary	58 (45)	170 (52)	95 (44)	43 (50)
College/University	14 (11)	45 (14)	41 (19)	9 (10)
Time between blood draw and diagnosis (years), mean (SD)	22.9 (7.8)	21.6 (8.7)	23.0 (8.3)	23.5 (8.3)
25‐hydroxyvitamin D(nmol/L), median (range)	42 (17–49)	63 (50–74.9)	85 (75–99.9)	109 (100–195)
DBP (µmol/L), median (range)	4.3 (2.2–8.6)	4.4 (1.8–7.4)	4.6 (3.0–8.0)	4.7 (3.9–9.6)
25(OH)D:DBP molar ratio (x 10^3^), median (range)	9.8 (4.0–17)	14 (8.0–31)	19 (11–31)	23 (13–36)
Cholesterol (mmol/L), median (range)	6.2 (1.6–11)	6.0 (3.4–9.4)	6.3 (3.4–11)	5.8 (3.9–11)
Triglycerides (mmol/L), median (range)	1.7 (0.3–10)	1.6 (0.48–7.6)	1.6 (0.34–12)	1.5 (0.39–6.1)

Abbreviations: 25(OH)D, 25‐hydroxyvitamin D; BMI, body mass index; DBP, Vitamin D‐binding protein; SD, standard deviation.

The results of the multivariable analyses investigating the association between 25(OH)D, DBP and 25(OH)D:DBP molar ratio and the risk of BC are presented in Table 3. The fully adjusted model (model 3) showed a borderline significant decreased risk of BC for optimal values of 25(OH)D (≥75 nmol/L) (HR 0.69, 95% CI 0.47–1.01, *p* = 0.054), and a significant decreased risk of high optimal values of 25(OH)D (≥100 nmol/L) (HR 0.35, 95% CI 0.19–0.64, *p* = 1.0·10^−3^), compared to the insufficient category (50–74 nmol/L). Moreover, deficient concentrations (<50 nmol/L) of 25(OH)D also showed a tendency of decreased BC risk, when compared to insufficient concentrations (HR 0.64, 95% CI 0.40–1.01, *p* = 0.055).

**TABLE 3:** Hazard ratio (HR) and 95% confidence interval (CI) of bladder cancer risk by levels of 25 (OH)D, DBP and 25(OH)D:DBP molar ratio

25OHD (nmol/L)	<50 (Deficient)	50–74 (Insufficient)	75–99 (Optimal)	≥100 (High Optimal)
*Case/control*	*61/68*	*181/143*	*102/115*	*34/52*
HR(95% CI)^a^	0.70 (0.46–1.07)	1 (ref)	0.70 (0.50–1.00)	0.48 (0.28–0.81)
HR(95% CI)^b^	0.62 (0.39–0.97)	1 (ref)	0.73 (0.50–1.06)	0.42 (0.24–0.74)
HR(95% CI)^c^	0.64 (0.40–1.01)	1 (ref)	0.69 (0.47–1.01)	0.35 (0.19–0.64)

DBP (umol/L)	<3.9	3.9–4.5	4.6–5.4	≥ 5.4
*Case/control*	*48/53*	*182/174*	*100/111*	*48/40*
HR(95% CI)^a^	0.86 (0.55–1.35)	1 (ref)	0.85 (0.57–1.26)	1.19 (0.69–2.07)
HR(95% CI)^b^	0.98(0.60–1.60)	1 (ref)	0.84 (0.55–1.27)	1.11(0.62–1.98)
HR(95% CI)^c^	0.90 (0.54–1.50)	1 (ref)	0.84 (0.56–1.28)	1.18 (0.66–2.13)

25OHD:DBP (*10^3^)	<11	11–16	17–21	≥22
*Case/control*	*63/63*	*193/158*	*84/101*	*38/56*
HR(95% CI)^a^	0.79 (0.51–1.24)	1 (ref)	0.67 (0.46–0.96)	0.52 (0.32–0.85)
HR(95% CI)^b^	0.68 (0.42–1.10)	1 (ref)	0.66 (0.44–0.97)	0.50 (0.29–0.82)

Abbreviations: 25(OH)D, 25‐hydroxyvitamin D; DBP, Vitamin D‐binding protein.

^a^ Conditioned on matching factors (age, sex, date, season, and county of blood draw).

^b^ Conditioned on matching factors (age, sex, date, season, and county of blood draw) and adjusted for BMI, physical activity, smoking (status and pack‐years), and education.

^c^ Conditioned on matching factors (age, sex, date, season, and county of blood draw) and adjusted for BMI, physical activity, smoking (status and pack‐years), education, DBP, and 25(OH)D, respectively.

No significant associations were observed between serum DBP concentrations and the risk of BC. However, an increasing 25(OH)D:DBP molar ratio, the estimate of free circulating 25(OH)D, was associated with decreased risk of BC. Compared to the molar ratio 11–16, a significantly decreased BC risk was found for molar ratio 17–21 (HR 0.66, 95% CI 0.44–0.97, *p* = 0.036) and molar ratio ≥22 (HR 0.50, CI% 0.29–0.82, *p* = 9.2·10^−3^).

**FIGURE 1:**
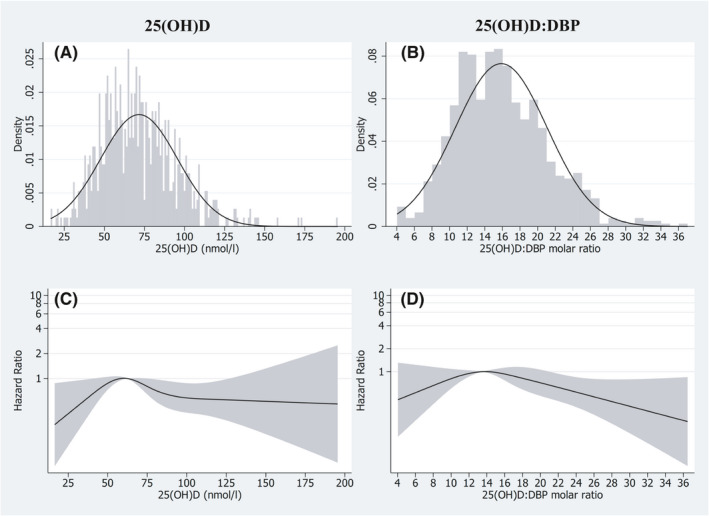
Histogram distribution of (A) 25(OH)D and (B) 25(OH)D:DBP molar ratio (x 10^3^). Restricted cubic splines displaying hazard ratios of bladder cancer risk with 95% confidence intervals according to (C) 25(OH)D and (D) 25(OH)D:DBP molar ratio (x 10^3^). For 25(OH)D reference was set to 62.5 nmol/L, *p*‐value for nonlinearity 0.0342. For 25(OH)D:DBP molar ratio (x10^3^) reference were set to 13.5 (molar ratio x 10^3^), *p*‐value for nonlinearity 0.0647. Both exposure risk curves are adjusted for matching factors (age, sex, time of blood draw), smoking (status and pack‐years) BMI, physical activity, and education

We present the distribution of 25(OH)D concentrations and 25(OH)D:DBP (Figure 1A and 1B) and their impact on BC risk on a continuous scale (Figure 1C and 1D). The distribution of the 25(OH)D concentrations is ranging from 16.5 to 195.4 nmol with a median value of 68.6 nmol/L and the HR of its effect on BC increased from deficient concentrations to the reference concentration (the median concentration of the reference category applied above), and thereafter decreased. The distribution of 25(OH)D:DBP molar ratio is ranging from 4.0–36.5 with a median value of 15.5. The HR increased from the lowest molar ratio category (<11) to the reference level of molar ratio of 13.5 (the median level of the reference category), and decreased thereafter. The spline models showed a better fit than the linear models for 25(OH)D and for 25(OH)D:DBP (*p* = 0.034 and *p* = 0.065, respectively).

The results for the stratified multivariable analyses are presented in Table 4. We did not observe statistically significant interactions between 25(OH)D and any of the variables examined, including smoking status, BMI, physical activity, and DBP (all *P* for interaction >0.10). However, the associations between high optimal concentrations and decreased BC risk was only statistically significant among current smokers (HR 0.39, 95% CI 0.19–0.81, *p* = 0.014), among individuals with BMI<25 kg/m^2^ (HR 0.39, 95% CI 0.19–0.78, *p* = 0.009) and individuals that are physical active (HR 0.44, 95% CI 0.25–0.80, *p* = 0.007). In addition, the association between deficient concentrations and decreased BC risk, was only statistical significant among individuals with BMI≥25 kg/m^2^ (HR 0.44, 95% CI 0.23–0.85, *p* = 0.013).

**TABLE 4:** Hazard ratio (HR) and 95% confidence interval (CI) of bladder cancer risk by concentrations of 25(OH)D, stratified by selected variables

		<50 (Deficient)	50–74 (Insufficient)	75–99 (Optimal)	≥100 (High Optimal)	*p‐value*
Smoking status						
Never smoker	*Case/control*	*(8/15)*	*(38/45)*	*(20/26)*	*(8/14)*	
	HR (95%CI)	0.53(0.18–1.53)	1 (ref)	0.91 (0.42–1.95)	0.61 (0.21–1.78)	
Former smoker	*Case/control*	*(9/23)*	*(39/45)*	*(25/38)*	*(7/13)*	
	HR (95%CI)	0.42 (0.16–1.05)	1 (ref)	0.84 (0.43–1.63)	0.56 (0.19–1.67)	
Current smoker	*Case/control*	*(44/30)*	*(104/53)*	*(57/51)*	*(19/25)*	
	HR (95%CI)	0.74 (0.39–1.39)	1 (ref)	0.55(0.32–0.96)	0.40 (0.19–0.83)	0.65
BMI						
<25 kg/m^2^	*Case/control*	*(31/29)*	*(97/78)*	*(60/72)*	*(20/36)*	
	HR (95%CI)	0.84 (0.45–1.59)	1 (ref)	0.68 (0.41–1.11)	0.40 (0.20–0.79)	
≥25 kg/m^2^	*Case/control*	*(30/39)*	*(84/65)*	*(42/43)*	*(14/16)*	
	HR (95%CI)	0.44 (0.23–0.85)	1 (ref)	0.82 (0.46–1.47)	0.46 (0.20–1.09)	0.37
Physical activity						
Sedentary	*Case/contol*	*(21/21)*	*(36/22)*	*(17/15)*	*(4/7)*	
	HR (95% CI)	0.54 (0.22–1.30)	1 (ref)	0.82 (0.31–2.16)	0.24 (0.06–1.03)	
Active	*Case/control*	*(40/47)*	*(145/121)*	*(85/100)*	*(30/45)*	
	HR (95% CI)	0.65 (0.38–1.11)	1 (ref)	0.71 (0.47–1.05)	0.44 (0.25–0.80)	0.78
DBP						
<median	*Case/control*	*(38/38)*	*(84/79)*	*(40/47)*	*(10/19)*	
	HR (95%CI)	0.86 (0.47–1.56)	1 (ref)	0.81 (0.46–1.41)	0.30 (0.12–0.77)	
>median	*Case/control*	*(23/30)*	*(97/64)*	*(62/68)*	*(24/33)*	
	HR (95%CI)	0.40 (0.20–0.82)	1 (ref)	0.64 (0.39–1.04)	0.44 (0.22–0.87)	0.30

Note

Conditioned on matching factors (age, sex, date, season, and county of blood draw). Additionally, adjusted for BMI, physical activity, smoking (status and pack‐years), and education.

Abbreviations: 25(OH)D, 25‐hydroxyvitamin D; DBP, Vitamin D‐binding protein.

## 4 DISCUSSION

In this population‐based case–control study, we found that pre‐diagnostic circulating 25(OH)D concentrations above high optimal levels (≥100 nmol/L) were associated with subsequent decreased BC risk when compared with insufficient concentrations (50–74 nmol/L). Moreover, free levels of 25(OH)D in circulation, the 25(OH)D:DBP molar ratio, was associated with decreased BC risk, when comparing high molar ratios (17–21 and ≥22) with the reference category (molar ratio 11–16). For both total and estimated free 25(OH)D, modeling the HR for the effect on BC risk by splines revealed that the effect was not linear, rather reversed u‐shaped, with the highest HR at 62.5 nmol/L and 13.5 molar ratio, respectively, and with a decrease thereafter. We did not find any association between DBP and BC risk. However, the association for free circulating 25(OH)D showed slightly larger effect with BC than total 25(OH)D concentrations.

The associated decreased risk of BC found for high circulating concentrations of 25(OH)D is consistent with the most recent meta‐analysis, which comprised two cohort and five case–control studies.^20^ The meta‐analysis showed that serum concentrations above 75 nmol/L were associated with a decreased risk of BC. Similarly, a pooling analysis of 17 cohorts recently reported that 25(OH)D concentrations above 75 nmol/L were associated with a decreased risk of colorectal cancer.^32^ Circulating concentrations of 25(OH)D above 75 nmol/L have been suggested as optimal to obtain full health benefits of vitamin D. However, there is no absolute agreement in what defines optimal 25(OH)D concentrations, especially not when it comes to cancer protective concentrations.^14^ In this study, concentrations above 100 nmol/L were associated with reduced BC risk, although levels above 75 nmol/L also showed a tendency of an association.

No association between DBP and BC risk was observed, which is in agreement with other studies.^23^ However, the estimate of free 25(OH)D showed a slightly stronger association with BC risk than total 25(OH)D concentrations, with decreasing risk in both categories of high molar ratios (17–21 and ≥22) compared to the reference category (molar ratio 11–16). This might indicate that the free 25(OH)D in circulation is a more relevant measure of 25(OH)D exposure with respect to BC risk than total 25(OH)D, which is supported by the free hormone hypothesis; that the biological activity is affected by the free circulating concentration.^33^ In our analysis, we used the 25(OH)D:DBP molar ratio as an estimation of free 25(OH)D. Errors due to an imperfect estimation would most likely not differ between case status. Thus, the actual measured free levels of 25(OH)D could be more strongly associated with BC than we observe in our analysis.

Preclinical studies have given mechanistic evidence for a protective role of vitamin D in BC development, demonstrating that the hormone form 1,25(OH)_2_D modulates gene transcription of antitumor genes, including genes with antiproliferative, anti‐invasive, and pro‐apoptotic properties.^34^ Animal and in vitro studies have shown in various models that 1,25(OH)_2_D suppresses BC development by reducing cell proliferation and stimulating apoptosis.^13^

On the other hand, high circulating levels of 25(OH)D are suggested to reflect a healthy lifestyle, and could thereby contribute to a protective association.^22^ According to our results, current smokers, those with elevated BMI and a lower physical activity levels, more frequently had low concentrations of 25(OH)D (Table 2). However, even though we incorporated solid information on various lifestyle factors into the analyses, they did not have an impact on our results. Despite no statistical significant interactions between lifestyle factors and 25(OH)D on the risk of BC, we observed statistical significant differences in the stratified analyses on the associations between high optimal concentrations and decreased BC risk among current smokers and individuals with normal BMI and high physical activity. The reason for not finding an interaction is possibly due to limited statistical power. Even though we were not able to detect any clear interaction from any of the lifestyle variables investigated, we cannot rule out that the protective associations we observe were related to lifestyle and/or residual confounding not sufficiently captured by our variables available.

Previous studies evaluating associations between 25(OH)D concentrations and BC risk have shown a dose‐dependent relationship, which might strengthen the evidence of causality (Hill's criterion).^18^, ^35^ We used spline functions to model the effect of vitamin D on BC risk, and found that the effect curve (HR) did not follow a linear relationship, rather a reversed u‐shape with the largest HR for insufficient concentrations (50–74 nmol/L). In particular, the group with deficient concentrations was not consistent with the assumption of linearity in the risk effect across 25(OH)D concentrations. One possible explanation is that the lowest concentrations are associated with other diseases or conditions that are inversely associated with BC risk. For instance high BMI is associated with low 25(OH)D concentrations, and have in some studies showed a tendency to be inversely related to BC risk.^7^, ^36^ In our analysis, when stratifying by BMI; a reduced BC risk for deficient 25(OH)D concentrations was only seen in the category of BMI ≥25.

A major limitation of our study is that serum samples for assessment of 25(OH)D and DBP were collected at one time point, which does not necessarily represent the individual's longitudinal vitamin D status relevant to cancer development. DBP is suggested to be relatively stable throughout adulthood.^37^ However, several factors are known to affect 25(OH)D levels over time, such as changes in diet, supplement use and time spent in the sun.^38^ Despite this, former studies have shown that circulating 25(OH)D measured several years apart were well correlated, although the correlation slightly declines over time.^39^, ^40^, ^41^, ^42^ Moreover, blood samples were collected in different periods, between 1972 and 2002, which could have affected the level and/or the quality of the samples differently. However, we accounted for the time point the blood sample was taken (including the season), by matching our cases and controls on the date and season of blood draw.

Our study has several strengths. First of all, we only sampled cases and controls without a cancer history, which together with our assessment of 25(OH)D and DBP in serum samples collected at least 5 years prior to the cancer diagnosis, reduces the risk of reverse causality. Also, we included detailed information on multiple potential confounding factors, including robust information about smoking history.

In conclusion, high serum levels of both total and estimated free 25(OH)D were associated with reduced risk of BC, when compared to insufficient levels. DBP was not associated with BC risk. We did not observe any impact of DBP or any of the studied lifestyle factors on the association between 25(OH)D and BC.

## CONFLICT OF INTEREST

The authors declare no competing interests.

## Supporting information

Appendix S1

## Data Availability

The data are available as presented in the paper. According to Norwegian legislation, our approvals to use the data for the current study do not allow us to distribute or make the data directly available to other parties.

## References

[1] Bray F , Ferlay J , Soerjomataram I , Siegel RL , Torre LA , Jemal A . Global cancer statistics 2018: GLOBOCAN estimates of incidence and mortality worldwide for 36 cancers in 185 countries. CA Cancer J Clin. 2018;68(6):394‐424. 30207593 10.3322/caac.21492

[2] Ploeg M , Aben KK , Kiemeney LA . The present and future burden of urinary bladder cancer in the world. World J Urol. 2009;27(3):289‐293. 19219610 10.1007/s00345-009-0383-3 PMC2694323

[3] Antoni S , Ferlay J , Soerjomataram I , Znaor A , Jemal A , Bray F . Bladder cancer incidence and mortality: a global overview and recent trends. Eur Urol. 2017;71(1):96‐108. 27370177 10.1016/j.eururo.2016.06.010

[4] Brown T , Slack R , Rushton L . British occupational cancer burden study G: occupational cancer in Britain. urinary tract cancers: bladder and kidney. Br J Cancer. 2012;107(Suppl 1):S76‐S84. 22710682 10.1038/bjc.2012.121 PMC3384013

[5] Burger M , Catto JWF , Dalbagni G , et al. Epidemiology and risk factors of urothelial bladder cancer. Eur Urol. 2013;63(2):234‐241. 22877502 10.1016/j.eururo.2012.07.033

[6] Rushton L , Bagga S , Bevan R , et al. Occupation and cancer in Britain. Br J Cancer. 2010;102(9):1428‐1437. 20424618 10.1038/sj.bjc.6605637 PMC2865752

[7] Hektoen HH , Robsahm TE , Andreassen BK , et al. Lifestyle associated factors and risk of urinary bladder cancer: A prospective cohort study from Norway. Cancer Med. 2020;9(12):4420‐4432. 32319230 10.1002/cam4.3060 PMC7300409

[8] Mondul AM , Weinstein SJ , Mannisto S , et al. Serum vitamin D and risk of bladder cancer. Cancer Res. 2010;70(22):9218‐9223. 20978193 10.1158/0008-5472.CAN-10-0985 PMC2982924

[9] Brinkman M , Zeegers MP . Nutrition, total fluid and bladder cancer. Scand J Urol Nephrol Suppl. 2008;218:25‐36. 10.1080/03008880802285073 18815914

[10] Bikle DD . Vitamin D metabolism, mechanism of action, and clinical applications. Chem Biol. 2014;21(3):319‐329. 24529992 10.1016/j.chembiol.2013.12.016 PMC3968073

[11] Bikle D . Nonclassic actions of vitamin D. J Clin Endocrinol Metab. 2009;94(1):26‐34. 18854395 10.1210/jc.2008-1454 PMC2630868

[12] Vanoirbeek E , Krishnan A , Eelen G , et al. The anti‐cancer and anti‐inflammatory actions of 1,25(OH)₂D₃. Best Pract Res Clin Endocrinol Metab. 2011;25(4):593‐604. 21872801 10.1016/j.beem.2011.05.001 PMC3164534

[13] Konety BR , Lavelle JP , Pirtskalaishvili G , et al. Effects of vitamin D (calcitriol) on transitional cell carcinoma of the bladder in vitro and in vivo. J Urol. 2001;165(1):253‐258. 11125420 10.1097/00005392-200101000-00074

[14] Holick MF . Vitamin D status: measurement, interpretation, and clinical application. Ann Epidemiol. 2009;19(2):73‐78. 18329892 10.1016/j.annepidem.2007.12.001 PMC2665033

[15] Bikle DD , Gee E , Halloran B , Kowalski MA , Ryzen E , Haddad JG . Assessment of the free fraction of 25‐hydroxyvitamin D in serum and its regulation by albumin and the vitamin D‐binding protein. J Clin Endocrinol Metab. 1986;63(4):954‐959. 3745408 10.1210/jcem-63-4-954

[16] Speeckaert M , Huang G , Delanghe JR , Taes YE . Biological and clinical aspects of the vitamin D binding protein (Gc‐globulin) and its polymorphism. Clin Chim Acta. 2006;372(1–2):33‐42. 16697362 10.1016/j.cca.2006.03.011

[17] Al‐oanzi ZH , Tuck SP , Raj N , et al. Assessment of vitamin D status in male osteoporosis. Clin Chem. 2006;52(2):248‐254. 16339300 10.1373/clinchem.2005.059568

[18] Grant WB . A review of the evidence supporting the Vitamin D‐cancer prevention hypothesis in 2017. Anticancer Res. 2018;38(2):1121‐1136. 29374749 10.21873/anticanres.12331

[19] Zhang H , Zhang H , Wen X , Zhang Y , Wei X , Liu T . Vitamin D deficiency and increased risk of bladder carcinoma: a meta‐analysis. Cell Physiol Biochem. 2015;37(5):1686‐1692. 26545152 10.1159/000438534

[20] Zhao Y , Chen C , Pan W , et al. Comparative efficacy of vitamin D status in reducing the risk of bladder cancer: A systematic review and network meta‐analysis. Nutrition. 2016;32(5):515‐523. 26822497 10.1016/j.nut.2015.10.023

[21] Brot C , Jorgensen NR , Sorensen OH . The influence of smoking on vitamin D status and calcium metabolism. Eur J Clin Nutr. 1999;53(12):920‐926. 10602348 10.1038/sj.ejcn.1600870

[22] Jääskeläinen T , Knekt P , Marniemi J , et al. Vitamin D status is associated with sociodemographic factors, lifestyle and metabolic health. Eur J Nutr. 2013;52(2):513‐525. 22538929 10.1007/s00394-012-0354-0

[23] Mondul AM , Weinstein SJ , Virtamo J , Albanes D . Influence of vitamin D binding protein on the association between circulating vitamin D and risk of bladder cancer. Br J Cancer. 2012;107(9):1589‐1594. 22990651 10.1038/bjc.2012.417 PMC3493763

[24] Hjerkind KV , Gislefoss RE , Tretli S , et al. Cohort profile update: The Janus Serum Bank Cohort in Norway. Int J Epidemiol. 2017;46(4):1101‐1102f. 28087783 10.1093/ije/dyw302

[25] Gislefoss RE , Stenehjem JS , Hektoen HH , et al. Vitamin D, obesity and leptin in relation to bladder cancer incidence and survival: prospective protocol study. BMJ Open. 2018;8(3):e019309. 10.1136/bmjopen-2017-019309 PMC5884376 29602840

[26] Larsen IK , Smastuen M , Johannesen TB , et al. Data quality at the cancer registry of Norway: an overview of comparability, completeness, validity and timeliness. Eur J Cancer. 2009;45(7):1218‐1231. 19091545 10.1016/j.ejca.2008.10.037

[27] Klingberg E , Oleröd G , Konar J , Petzold M , Hammarsten O . Seasonal variations in serum 25‐hydroxy vitamin D levels in a Swedish cohort. Endocrine. 2015;49(3):800‐808. 25681052 10.1007/s12020-015-0548-3 PMC4512566

[28] Lips P , Cashman KD , Lamberg‐Allardt C , et al. Current vitamin D status in European and Middle East countries and strategies to prevent vitamin D deficiency: a position statement of the European calcified tissue society. Eur J Endocrinol. 2019;180(4):P23‐P54. 30721133 10.1530/EJE-18-0736

[29] Hadkhale K , MacLeod J , Demers PA , et al. Occupational variation in incidence of bladder cancer: a comparison of population‐representative cohorts from Nordic countries and Canada. BMJ Open. 2017;7(8):e016538. 10.1136/bmjopen-2017-016538 PMC5629726 28780557

[30] Cogliano VJ , Baan R , Straif K , et al. Preventable exposures associated with human cancers. J Natl Cancer Inst. 2011;103(24):1827‐1839. 22158127 10.1093/jnci/djr483 PMC3243677

[31] Harrell FE . General Aspects of Fitting Regression Models. Regression Modeling Strategies: With Applications to Linear Models, Logistic and Ordinal Regression, and Survival, Analysis. edn. Cham: Springer International Publishing; 2015:13‐44.

[32] McCullough ML , Zoltick ES , Weinstein SJ , et al. Circulating vitamin D and colorectal cancer risk: an international pooling project of 17 cohorts. J Natl Cancer Inst. 2019;111(2):158‐169. 29912394 10.1093/jnci/djy087 PMC6376911

[33] Mendel CM . The free hormone hypothesis: a physiologically based mathematical model*. Endocr Rev. 1989;10(3):232‐274. 2673754 10.1210/edrv-10-3-232

[34] Fleet JC , DeSmet M , Johnson R , Li Y . Vitamin D and cancer: a review of molecular mechanisms. Biochem J. 2012;441(1):61‐76. 22168439 10.1042/BJ20110744 PMC4572477

[35] Hill AB . The environment and disease: association or Causation? Proc R Soc Med. 1965;58:295‐300. 14283879 10.1177/003591576505800503 PMC1898525

[36] Vanlint S . Vitamin D and obesity. Nutrients. 2013;5(3):949‐956. 23519290 10.3390/nu5030949 PMC3705328

[37] Haddad JG . Plasma vitamin D‐binding protein (Gc‐globulin): multiple tasks. J Steroid Biochem Mol Biol. 1995;53(1–6):579‐582. 7626513 10.1016/0960-0760(95)00104-8

[38] McKenna MJ , Murray BF , O'Keane M , Kilbane MT . Rising trend in vitamin D status from 1993 to 2013: dual concerns for the future. Endocrine Connections. 2015;4(3):163‐171. 26034120 10.1530/EC-15-0037 PMC4496526

[39] Hofmann JN , Yu K , Horst RL , Hayes RB , Purdue MP . Long‐term variation in serum 25‐hydroxyvitamin D concentration among participants in the prostate, lung, colorectal, and ovarian cancer screening trial. Cancer Epidemiol Biomarkers Prev. 2010;19(4):927‐931. 20332255 10.1158/1055-9965.EPI-09-1121 PMC2857993

[40] Jorde R , Sneve M , Hutchinson M , Emaus N , Figenschau Y , Grimnes G . Tracking of serum 25‐hydroxyvitamin D levels during 14 years in a population‐based study and during 12 months in an intervention study. Am J Epidemiol. 2010;171(8):903‐908. 20219763 10.1093/aje/kwq005

[41] McKibben RA , Zhao D , Lutsey PL , et al. Factors associated with change in 25‐hydroxyvitamin d levels over longitudinal follow‐up in the ARIC Study. J Clin Endocrinol Metab. 2016;101(1):33‐43. 26509869 10.1210/jc.2015-1711 PMC4701839

[42] Kubiak J , Kamycheva E , Jorde R . Tracking of serum 25‐hydroxyvitamin D during 21 years. Eur J Clin Nutr. 2020. 10.1038/s41430-020-00814-0 33311556

