# Supplementary material for: Vitamin D and Vitamin D‐binding protein and risk of bladder cancer: A nested case‐control study in the Norwegian Janus Serum Bank Cohort
Source: Cancer Med. 2021 Jun 3;10(12):4107–16. doi: 10.1002/cam4.3960 (PMC8209600; doi:10.1002/cam4.3960)
Supplement: Supplementary file 1 — Appendix S1 [file CAM4-10-4107-s001.docx]

**Supplementary material**

**Summary**

1. Overview of study design (Figure S1)
2. Season adjustment (Figure S2)
3. Sensitivity analysis (Table S1, Figure S3)
4. **Overview of study design**

**Figure S1**

**
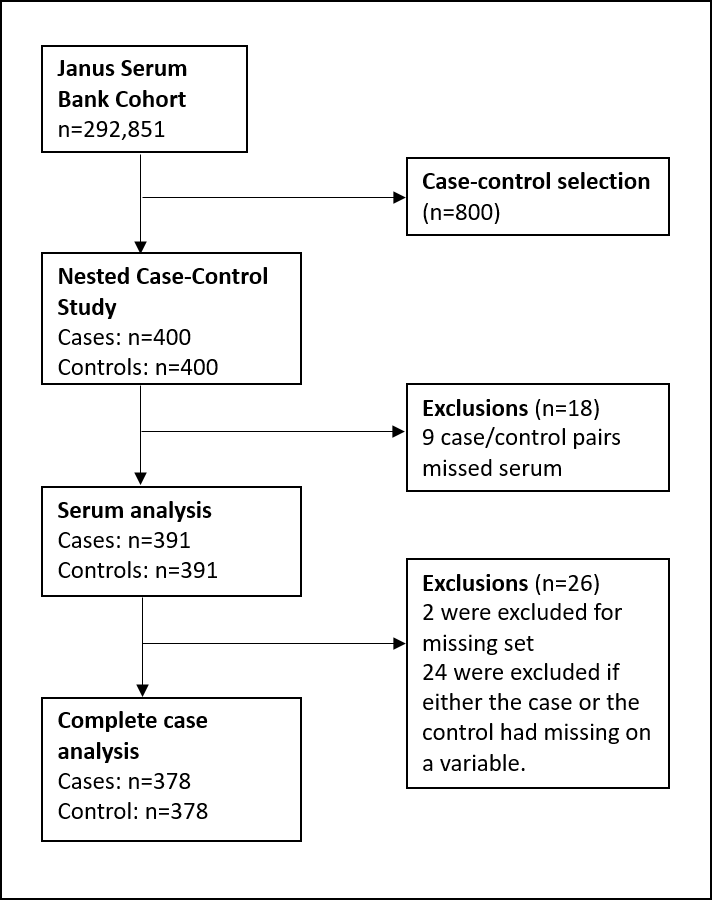
**

**Figure S1.** Overview of study design (selection of cases and controls) and exclusions.

1. **Season adjustment**

We modelled the seasonal variation in our study sample by performing a least square fit of a sine function to the measured concentrations of 25(OH)D versus date of blood draw.

$$y(d)=y0+amplitude*sin\left( \frac{2\pi}{365}*d+phase shift \right)$$

*Y (d)* is the 25(OH)D concentration at *d* (number of days from January 1st). *y0* represents the mean level of the sine curve, and thus an estimate of the annual mean of 25(OH)D of the study sample. The *amplitude* represents the seasonal variation and is the maximal deviation from y0. The *phase shift* is the translation along the x-axis. An amplitude of 14.49, y0=71.93 and phase shift=3.97 was obtained providing a maximum 25(OH)D concentration in mid august (14th) and a minimum in the end of February (29th). To adjust measured 25(OH)D values for seasonal variation, we calculated the individual deviations from the fitted sine curve to the annual mean of the study sample, thereby calculating an annual value for each participant, which mean solving the equation for y0 for each individual. The seasonal variation of the measured 25(OH)D concentrations, by month of blood draw, and the modelled sine function is shown in figure S2.

**Figure S2**

**

**

**Figure S2** Seasonal variations of 25(OH)D concentrations among the whole study population, by month of blood draw. The box plot shows the median 25(OH)D concentration as a horizontal line and encompass the 25^th^ and 75^th^ percentiles, The solid line represents the predicted geometric mean concentrations given by date of blood draw, which was modelled as a sine function.

1. **Sensitivity analysis**

To assess the influence of extreme values of 25(OH)D and DBP concentrations on the results, we performed sensitivity analyses excluding values below the 2.5 percentile or above the 97.5 percentile. The results are shown in Table S1 and Figure S3.

**Table S1** Hazard ratio (HR) and 95% confidence interval (CI) of bladder cancer risk by levels of 25 (OH)D, DBP and 25(OH)D:DBP molar ratio, excluding values below the 2.5 percentile or above the 97.5 percentile.

| **25OHD (nmol/L)** | **<50 (Deficient)** | **50–74 (Insufficient)** | **75–99 (Optimal)** | **≥100 (High Optimal)** |
| --- | --- | --- | --- | --- |
| *Case/control* | *52/61* | *182/146* | *104/117* | *44/24* |
| HR(95% CI)^1^ | 0.53 (0.30–0.88) | 1 (ref) | 0.73 (0.48–1.10) | 0.29 (0.14–0.60) |
| **DBP (umol/L)** | **<3.9** | **3.9–4.5** | **4.6–5.4** | **≥ 5.4** |
| *Case/control* | *54/61* | *167/159* | *94/104* | *45/47* |
| HR(95% CI)^1^ | 0.85 (0.52–1.38) | 1 (ref) | 0.85 (0.55–1.32) | 1.03 (0.56–1.88) |
| **25OHD:DBP (*10^3^)** | **<11** | **11–16** | **17–21** | **≥22** |
| *Case/control* | *50/57* | *184/155* | *81/99* | *28/46* |
| HR(95% CI)^1^ | 0.58 (0.34–0.99) | 1 (ref) | 0.67 (0.44–1.03) | 0.50 (0.27–0.95) |

^1^Conditioned on matching factors (age, sex, time of blood draw). Additionally, adjusted for BMI, smoking (status and packyears), education and mutually adjusted for DBP and 25OHD


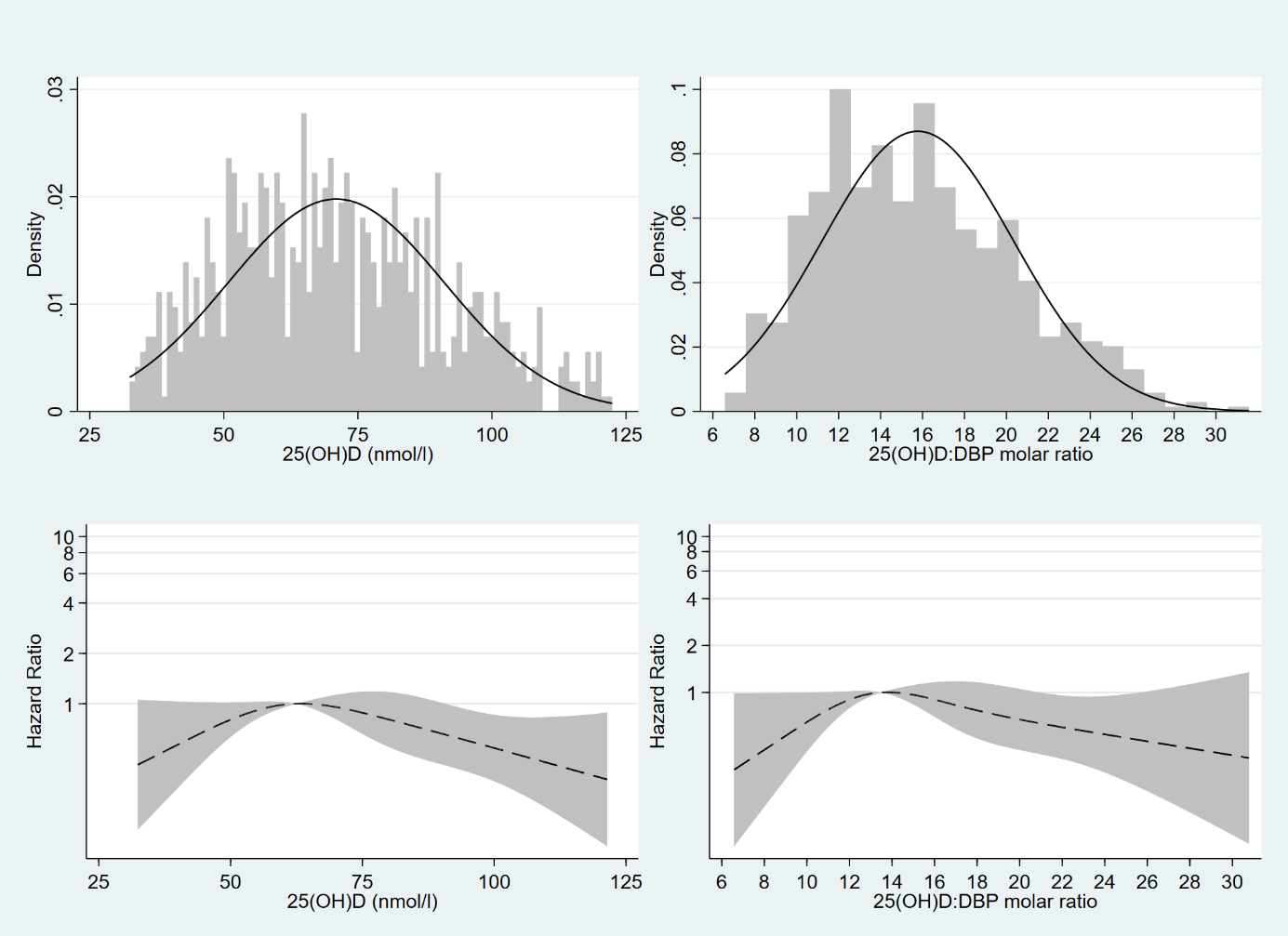


D

C

A

B

**Figure S3** Histogram distribution of A) 25(OH)D and B) 25(OH)D:DBP molar ratio (x 10^3^). Restricted cubic splines displaying hazard ratios of bladder cancer risk with 95% confidence intervals according to C) 25(OH)D and D) 25(OH)D:DBP molar ratio (x 10^3^) . For 25(OH)D reference was set to 62.5 nmol/L, *P*-value for non-linearity 0.0279. For 25(OH)D:DBP molar ratio (x10^3^) reference were set to 13.5 (molar ratio x 10^3^), *P-*value for non-linearity 0.0488. Both exposure risk curves are adjusted for matching factors (age, sex, time of blood draw) and smoking (status and pack-years) BMI, physical activity and education.
